# Urban Lighting Research Transdisciplinary Framework—A Collaborative Process with Lighting Professionals

**DOI:** 10.3390/ijerph18020624

**Published:** 2021-01-13

**Authors:** Catherine Pérez Vega, Karolina M. Zielinska-Dabkowska, Franz Hölker

**Affiliations:** 1 Leibniz-Institute of Freshwater Ecology and Inland Fisheries, Müggelseedamm 310, 12587 Berlin, Germany; perez.vega@igb-berlin.de (C.P.V.); hoelker@igb-berlin.de (F.H.); 2 Department of Biology, Chemistry, and Pharmacy, Institute of Biology, Freie Universität Berlin, 14195 Berlin, Germany; 3 Faculty of Architecture and Design, Hochschule Wismar University of Applied Sciences Technology, Business and Design, 23966 Wismar, Germany; 4 GUT Light Lab, Faculty of Architecture, Gdansk University of Technology (GUT), 80-233 Gdansk, Poland

**Keywords:** light pollution, ecological light pollution, sustainable lighting, urban planning, lighting professionals, ALAN researchers, urban lighting research, transdisciplinary research

## Abstract

Over the past decades, lighting professionals have influenced the experience of the night by brightly illuminating streets, buildings, skylines, and landscapes 24/7. When this became the accepted norm, a dual perspective on night-time was shaped and the visual enjoyment of visitors after dusk was prioritized over natural nightscapes (nocturnal landscapes). During this time, researchers of artificial light at night (ALAN) observed and reported a gradual increase in unnatural brightness and a shift in color of the night-time environment. As a consequence, ALAN has been identified as a relevant pollutant of aquatic and terrestrial habitats, and an environmental stressor, which may adversely affect a wide range of organisms, from micro-organisms to humans. Unfortunately, lighting professionals and ALAN researchers usually attempt to solve today’s sustainable urban lighting problems distinctive to their fields of study, without a dialogue between research and practice. Therefore, in order to translate research knowledge as an applicable solution for the lighting practice and to minimize the impact on the environment, a collaborative framework involving a transdisciplinary process with lighting professionals is crucial to potentially bring the practice, research, production, decision-making, and planning closer to each other. This paper presents a framework to help reduce the existing gap of knowledge, because appropriate lighting applications depend upon it. Access to less light polluted nightscapes in urban environments is just as important as access to unpolluted water, food, and air. This call for action towards sustainable urban lighting should be included in future lighting policies to solve the urgent environmental and health challenges facing our world.

## 1. Introduction

Today, most cities and towns have a prolific network of artificial light at night (ALAN) that illuminates traffic and pedestrian routes, as well as buildings and landscapes, not only for visibility but also to provide visitors and residents with visual enjoyment and entertainment after dusk [1,2].

Over time, as the network of ALAN expanded from urban to peri-urban environments towards rural landscapes [3], the globe has endured the spread of unnatural brightness and vivid colors of light at night, which is today encroaching into new territories that were previously unlit [4].

Over the past two decades, night-time studies have reported evidence on the presence of ALAN as an unintended form of anthropogenic pollution, known as light pollution (LP) [5,6] and ecological light pollution (ELP) [7] which has significantly increased over the years [4,8,9]. It has been described as the inappropriate application and management of artificial light sources and luminaires cohered to the rapid development of urbanization, which leads to the unnecessary and undesired emissions of ALAN across landscapes [10,11]. The latest research on the environmental impact of ALAN indicates that night-time environments in aquatic and terrestrial ecosystems are increasingly impacted by light pollution [12,13,14,15]. ALAN has become a stressor of natural cycles and biological processes that potentially threatens biodiversity [16,17,18,19], and biological rhythms in humans and nature [20,21,22,23,24]. Moreover, an induced suppression of melatonin, the night hormone, has been reported in various vertebrate species [25] even when exposed to comparative low skyglow light levels. Although the lighting practice has gained awareness of the concept of light pollution, [26,27] as the result of incorrectly managed properties of artificial lighting, the challenge remains to translate research notions beyond the known concept of light as a pollutant to the actual planning and design of lighting schemes that provide appropriate illuminance levels for visibility, whilst also protecting the natural night-time environment [11,28,29,30,31].

## 2. Challenges for the Practice

Practical solutions to address the rapid rise of ALAN and minimize its adverse effects have become challenging and complicated to implement due to various reasons.

### 2.1. Reasons to Consider

Firstly, the prompt development of urbanization encouraged the planning, designing, and application of technical lighting parameters that centered on users [32]. These solutions corresponded to photopic vision during the day [33], which is the visual sensitivity and ability to see when exposed to bright environments [34]. Eventually, the simulation of daylight conditions for night-time environments was implemented as a means to translate a sense of visual understanding across night-time landscapes. The lighting practice recognized the application of artificial lighting as a way to provide safe passage for pedestrians and to allow vehicles to circulate at night [35,36], but environmental concerns were usually ignored. This use of artificial lighting soon became an environmental concern because luminaires were continuously operated from dusk to dawn, which impaired circadian rhythms (set by location-specific natural day and night cycles). It also created a continuous period of brightness with unnatural colors of light at night, emitted from artificial lighting across landscapes.

Secondly, potential environmental problems related to, for example, biodiversity and the protection of natural nightscapes have rarely been discussed in the lighting practice [37,38,39]. This is mostly owing to the fact that to understand the impact of artificial lighting technologies on biodiversity, at least a basic disciplinary knowledge of ecology is required.

Thirdly, in recent years, the lighting industry and its practice continues to promote the replacement of conventional lighting technologies (e.g., high- and low-pressure sodium—LPS and HPS) to solid-state lighting applications (with the focus on light-emitting diodes—LEDs). LEDs are considered a “sustainable lighting solution” that delivers higher efficacy at a lower cost. However, saving energy costs does not solve the problem of increased emissions of light at night (rebound effects, e.g., [4,40]) or luminaires that direct undesired and unnecessary light upwards, without considering the impact of downward emissions towards aquatic and terrestrial environments, whilst also operating luminaires continuously from dusk to dawn.

Lastly, in regards to the temporal and spatial emission patterns of ALAN, the current discussion about light pollution and available lighting standards and guidelines, both focus solely on the protection of the night sky [41,42,43,44]. However, the evaluation of obtrusive light and the approval of lighting installations as an enforced practice rarely occurs [26]. Although in some countries there are existing regulatory frameworks such as guidelines, procedures, standards and codes, or legal acts relevant for urban lighting and light pollution (Table 1), unfortunately there are no globally established design and technical parameters available for lighting projects that aim to be respectful to ecology and the natural environment. Most often, the lighting design process lacks an Ecological Impact Assessment in Feasibility Study, which may vary from one country to the next [45,46]. Additionally, lighting professionals and ALAN researchers usually attempt to overcome problems particular to their field of expertise, with insufficient dialogue across research and practice disciplines. The vocabulary and know-how of both domains differ, as they address issues that are specific and relevant to their field of study.

Consequently, the adoption of collaborative practices via the exchange of vocabulary, expertise, and knowledge rarely occurs, even if both lighting practitioners and ALAN researchers have an increasing interest in collaboration [2,49,50,51].

### 2.2. Towards Inclusive, Collaborative, and Transdisciplinary Lighting Research and Practice

In 2010, ALAN researchers had already emphasized that a transdisciplinary research agenda can potentially favor the development of regulations and guidelines that meet environmental needs and the demands of modern societies [8]. Interestingly, lighting professionals were often not considered back then as part of the actors involved. However, today, lighting professionals are judged to be crucial team members who can introduce new ways of thinking about research problems. They can also provide essential research inputs based on their practical knowledge of artificial lighting.

As the dialogue between lighting professionals and ALAN researchers remains insufficient to translate the acquired data as knowledge into the practice domain, it is of great significance to improve the application of artificial light to minimize the impact of light pollution on the environment via a more efficient and effective collaboration between researchers, lighting practitioners, policymakers, and society in the context of urban lighting. With this paper, we aim to provide a brief insight into the perspectives of the lighting practice and research on night-time studies, we explain the actors involved in each domain, while also offering potential platforms to exchange and transfer knowledge, and we make a call for action on an improved transdisciplinary approach. The proposed collaboration framework has the purpose of bringing those involved in practice, research, production inputs, planning, and policy-making domains all closer in order to conduct and coordinate the organizational structure in which information flows. This transdisciplinary teamwork may serve to translate and convey research knowledge between lighting professionals and ALAN researchers, to better provide healthier and more sustainable urban settings, which are inclusive and protective of the night.

## 3. Development of the Dual Perspective of the Night

**Figure 1:**
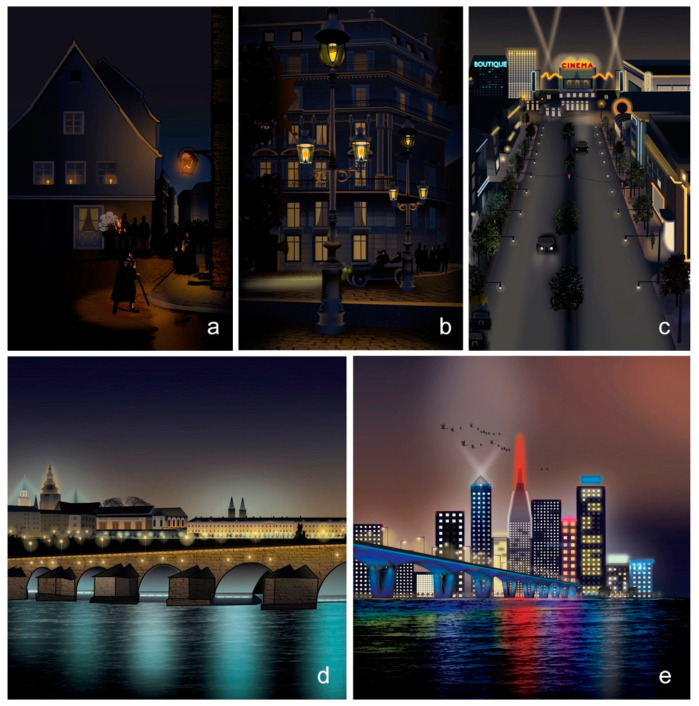
The evolution of urban illumination over time, illustrating the varied approaches of artificial lighting practice used to deliver illuminated night-time scenarios in built and natural landscapes. In the beginning, there were (**a**) relatively dark pedestrian pathways with dappled pools of light at street intersections and soupçons of light washing the first-floor façade of important buildings mainly used by citizens at night. Later, (**b**) robust iron poles with lanterns were applied to luster streets, roads, and paths. Then, (**c**) skyscrapers and tall buildings acquired illuminated elements for advertisement with vertically illuminated façades that reveal the structure of buildings. In the following years, (**d**) functional and decorative lighting co-existed in the same urban realm to make cities and towns functional and aesthetically pleasing at night. In recent years, (**e**) functional lighting for pedestrian and vehicular circulation and decorative lighting for skyscrapers, buildings, monuments, and landmarks have presented a changing luminosity and color condition with shifting shapes, patterns of shadow and light, along with videos projected onto buildings so they become illuminated canvases at night. Source: authors’ own work.

In medieval times, most cities and towns were in darkness after dusk so additional illumination via lit candles and torches was used for wayfinding along routes [52]. Since then, the practice of lighting incorporated varied approaches to deliver illuminated night-time scenarios in built and natural environments (Figure 1).

Dark pathways were dappled with pools of artificial light in core key locations (e.g., pedestrian street intersections or the façades of important buildings). Areas were illuminated with light rich in long wavelengths with warmer correlated color temperatures (CCT) at approximately 1800–2200 K [52]. The light distribution of these light sources was also closer to the ground and restricted to areas of common passage. Night-time illumination had not yet infiltrated terrestrial and aquatic landscapes as portrayed in the 1879 impressionist oil painting *Walk with lanterns* by Ilya Repin [53]. Skyglow was also not considered a problem, as cities still remained relatively dark at night (Figure 1a).

Later, gas lighting technology and the emergence of electric lighting provided the means to establish urban lighting systems with multiple fixed lanterns in poles to increase illuminance parameters and to deliver horizontal homogeneous illumination that did not occur in medieval times. An increased number of lanterns per fixed pole were applied in streets, roads, and paths to enable pedestrian and vehicular circulation. The spectral power distribution (SPD) of these point sources was considered rich in long wavelengths with a correlated color temperature that increased from approximately 2000 up to 2700 K. The introduction of these forms of urban artificial lighting producing low light levels were expected to attract, for example, insects in the vicinity of the applied sources, “*Wie Motten um das Licht*”—*like moths around a flame*, as mentioned in the song *Falling in love again* by Marlene Dietrich [54]. Urban areas began to show the first traces of skyglow in cities and towns (Figure 1b).

Then, in addition to the horizontal illuminance parameters, artificial light was used as a medium on the vertical surface of tall buildings and skyscrapers to sculpt volumes for three-dimensional spaces. During the 20th century, multiple techniques emerged that included the uplighting of building façades as well as the use of searchlights, which are high-intensity electric sources of light used for finding objects in the distance at night. Searchlights were positioned to direct emissions of light towards the sky [55]. Furthermore, decorative advertising lighting systems were used to illuminate the top of buildings with marquee signs and letterings. Unfortunately, the position and direction of point sources used to illuminate the vertical plane directed light towards the sky. They also delivered higher illuminance parameters when compared with lighting systems used in previous years. Such light sources were present in illuminated parks, exposing the leaves of trees and plants to the emissions of light; as observed and reported in 1975, ALAN was considered to affect the flowering state in varied flowering plants [56].

Additionally, the beams of floodlights on the roofs of theatres and cinemas became iconic urban illuminated elements that were observed to attract migrating birds at night, as shown at the Eddystone lighthouse illustrated in 1912 [57]. In comparison to previous years, artificial lighting began to feature broad ranges of SPD from approximately 400 to 700 nm, with richness in short wavelengths of light and less red wavelengths. This was coupled with an increase in the range of CCT from approximately 2000 K up to 5000 K [58,59]. The combination of techniques and applied light sources resulted in an unintended diffused luminous dome, visible over densely populated areas at night. This man-made effect is called skyglow. The use of light sources with a broad SPD rich in short wavelengths has allowed illuminated landscapes at night to appear as they would during the day. Artificial light at night became an artificial accoutrement of nightscapes and a convincing cultural and social excuse to construct and promote over-illuminated cities that unintentionally neglect the loss of the night [60]. Population growth, the exponential increase in illumination per capita, and the increased number of point sources and numerous applied techniques across landscapes have all presented unforeseen consequences, reported at the end of 1980s with the first research articles on light as an anthropogenic, artificial, and detrimental component. The aim of this research was to raise awareness about the problem in the form of skyglow and to also help reduce light pollution from urban and rural environments [5,6] (Figure 1c).

It soon became strategic to use new lighting techniques for the scene setting of local historical landmarks to change the overall perception of urban built environments. At the end of the 20th century, applied lighting techniques mainly focused on engaging the user with the encountered space at night with higher illuminance parameters, varied correlated color temperatures, and spectral power distributions that ranged across the visible spectrum, shadows, and the duality of brightness and darkness [61,62,63,64,65]. The upward emission of artificial lighting coupled with higher levels of brightness has resulted in significant light pollution across ecosystems (e.g., night-time illuminated bridges, which emit artificial light towards aquatic ecosystems and are known to negatively affect migrating salmon fish [66], and uplit trees and green areas in parks and public gardens, which may adversely alter other organisms, such as insects [16,67]). Night-time illumination soon reached areas that were once unlit (Figure 1d). These approaches typically defined lighting practices that focused on the role of night-time illumination in an urban context to engage the user with the various spaces they used at night [68], and the detrimental impact of artificial lighting on humans and nature was not yet considered by lighting experts.

Meanwhile, ALAN researchers focused on gathering evidence of artificial light as a long-term phenomenon and pollutant of night-time environments [69]. This includes the founding notions and observations of the sky by night. Since the 1900s, the earliest measurements of photon emissions produced per capital demonstrated that cities were under an artificially bright night sky [70].

Astronomers and astrophysicists reported a noticeable reduction in the visibility of the naturally dark sky due to an increase in illuminance and the use of unnaturally vivid colors of light at night in densely populated areas [71,72]. Over the years, the application of artificial lighting with upward emissions of light and higher levels of brightness resulted in significant light pollution across landscapes.

Unfortunately, opportunities to acquire this new knowledge to understand the consequences of the inappropriate use of ALAN was scarce, as the notion that ALAN is a pollutant was still being explored by ALAN researchers.

Moreover, at the beginning of the 21st century, there was a distinct lack of knowledge about the numerous properties of artificial lighting, which are now known to be pollutants. There was also a lack of communication, which was needed in order to translate research into the practice of lighting.

New technological developments enabled multiple techniques to emerge. This included dynamic artificial lighting with changes in brightness, color temperatures, colored light, and shadow patterns, as well as the projection of videos onto buildings so they became illuminated canvases [61,62,63,64,65]. The increased application of artificial lighting came at the expense of the natural night-time environment. ALAN researchers estimated that there was a higher variability of artificial lighting conditions across landscapes, such as significantly increased levels of brightness [4,19] as well as the widespread use of unnatural colors, which is considered to affect varied organisms [14]. Upward illumination towards the sky, such as the 9/11 Memorial—*Tribute in Light* [73], has been reported to affect migrating birds [74]; another example is the bat colonies in various illuminated churches across Sweden, where bats have been observed to change their flight trajectories to avoid illuminated areas, which can potentially affect the choice of corridors during flight and may fragment the selection of foraging areas for bats [75] (Figure 1e).

Previously unlit areas at night were now being illuminated, (e.g., illuminated bridges that disturb the ecology of rivers and bodies of water, as well as skyscrapers and tall building that emit light towards the sky, which harms birdlife and insects). During recent years, light pollution and ecological light pollution have been diligently investigated via scientific research to broaden the understanding of the impact of night-time lighting applications on humans and nature [7,20,21,22,24,25,76].

Table 2 provides a description of the evolution of outdoor lighting chronologically over time.

In the first decades of the 21st century, ALAN researchers have provided evidence on night-time illumination as a pollutant that disrupts biological rhythms in humans and nature [9,80,81] and have estimated an increase in LP of 2–6% per year [4,19]. Additionally, environmental researchers have reported that LP and ELP inhibit crucial day and night-time cycles across taxa, since 30% of all vertebrates and more than 60% of all invertebrates have visual sensitivities attuned to natural low light levels, which involves the ambient illuminance of lunar cycles and starlight in the night-time environment [19]. An extensive body of empirical evidence has identified potential behavioral and physiological changes in responses induced by properties of ALAN across taxa; this includes terrestrial organisms such as bats [82], birds [83], and insects [16,84], micro-organisms, and aquatic species exposed to artificial lighting near waterfronts, bridges, rivers, and lakes [13,85,86].

Eventually, a dual perspective of the night was formed as both lighting professionals and ALAN researchers developed individual outlooks focused on ALAN. This caused a no-win/Gordian Knot situation [87,88,89] that challenged the research and lighting practice due to different approaches and limitations. For the majority of the lighting practice, the method for lighting technologies involved expertise that mainly focused on delivering the user visual accessibility for visited areas at night, and beyond that, a sense of connection to urban night-time environments. Meanwhile, ALAN researchers presented expertise focused on the study of an unnatural component, such as artificial light and its properties and the resulting adverse effects across night-time environments, and this included the widespread use of ALAN causing skyglow: a global phenomenon responsible for the loss of the night (e.g., [90,91]).

The actions against light pollution and the improper management of lighting applications are not necessarily a confrontation between ALAN researchers and lighting professionals [39,92], but rather an attempt to start a transdisciplinary collaboration between experts, which includes best practice and sustainable lighting applications that serve society’s interests while respecting ecology [93]. An example of a transdisciplinary collaboration between environmental and lighting experts occurred in the city of Berlin, Germany, in 2011. This involved a Lighting Advisory Board, which included lighting and environmental experts in collaboration with the Senate Department for Urban Development of the city of Berlin, to conceptualize the urban lighting masterplan for the city of Berlin (Stadtbild Berlin–Lichtkonzept) (Senatsverwaltung für Stadtentwicklung und Umwelt, 2011). The lighting masterplan concept portrays the city as an economic center and provides citizens with a sense of safety and security whilst enhancing the allure of the city with lighting technologies that minimize the impact of light upon ecology at night. 

The conceptualization of a transdisciplinary masterplan for the city of Berlin demonstrated one of the first attempts towards openness for an environmental lighting perspective during a time when the scientific evidence on the impact of ALAN across urban and natural environments was still unclear. Ten years later, scientific research on the topic has substantially increased.

## 4. Bridging Domains in the Collaborative Process

It is essential to understand that any successful collaborative process involves a philosophy of empathic interactions that acknowledge the diverse languages, perspectives, skillsets, backgrounds, and expertise of each domain [94].

The collaborative nature can potentially occur only as a dynamic interaction driven by a shared interest as an emboldened modality [95], aimed at reducing the impact of artificial lighting on the environment with solutions that address different night-time conditions (e.g., cloudy nights or clear sky conditions) and different nightscape contexts.

In order to communicate crucial knowledge about ALAN (as a potential pollutant, while also recognizing that ALAN is a tool to implement visual accessibility for users at night), the lingua franca becomes a strategic asset and a frame of reference to communicate the knowledge acquired by experts, the contrasting perspectives, and the platforms for the transmission of evidence into practical solutions [96].

The language used to transfer information may serve to bridge the existing gaps between practice, research, production, planning, and policy-making domains and may enrich a shared empathic comprehension of the research questions and problems that each domain is focused upon. Over the past century, the field of lighting design/technology and the field of biology/environmental sciences have both developed key terms independently of each other, in regards to the study of the night-time environment. However, the interpretation of these definitions varies.

Lighting professionals define properties of artificial lighting for the built environment that consider human vision focused on the perception of brightness, contrasts, and colors in objects under lighting conditions. Whereas, light pollution researchers and environmental experts in biology, ecology, chronobiology, and light pollution developed a vocabulary that identifies and describes the effect of natural and artificial light on biological processes, living organisms, and ecosystems, as well as how environments and organisms affect each other. The International Commission on Illumination (CIE)—the international authority on light and lighting—recently created an online international lighting vocabulary [97], but terms such as “ALAN” and “ecological light pollution” have not been defined [98]. The CIE Technical Committee TC 4-61 has yet to produce a report and propose additions to the missing definitions to reduce this communication gap between ALAN researchers and lighting professionals.

Furthermore, the application of light can also greatly vary among scholars, experts, and professionals in these fields. Lighting professionals see it positively for the visual perception of built and natural landscapes at night and the added value it gives cities and towns, which supports their visual appearance and so forth, however, this is often without consideration of the possible consequences of added light. Whereas, environmental experts consider artificial light as a component of society in need of careful management to reduce the current negative burden on biodiversity and the natural environment.

It is important to stress that accessibility to knowledge in both domains requires on-going interactions between the participants involved in order to build responsiveness and potentially address current and emerging issues [99]. If guided incorrectly, it may amplify the existing gap between lighting professionals and ALAN researchers, stoking existing tension that may lead to uncertainties that disrupt the dynamic and co-learning circumstances for each domain.

For instance, it is crucial to start with a dialogue about the problems that each field of study addresses. Lighting professionals can attempt to answer the following questions: How to design lighting schemes in urban environments that are based on research? What lighting technology to implement? How to apply it for a specific project application?

Meanwhile, ALAN researchers can consider the following questions in their day-to-day work: Based on the requirements of the lighting practice, what needs to be researched? What properties in artificial lighting technologies are considered a light stressor? When is it considered a stressor? [8,65].

The background and know-how of lighting professionals and ALAN researchers may create new approaches and enrich techniques to achieve goals beyond the individual narrative of each domain. The dialogue between experts may serve as a negotiating process to address existing differences while creating a new vision to answer the question of how to translate research knowledge to be applicable for the lighting practice.

Educational awareness on LP has also taken place by means of nonprofit organizations, networks, and conferences.

The International Dark-Sky Association (IDA) [100], the Australasian Dark Sky Alliance [101], and other nonprofit organizations [48] educate communities and government officials on the protection of night skies and biologically/ecologically responsible outdoor lighting.

Additionally, the interdisciplinary and transdisciplinary network EU-COST Action “Loss of the Night Network” (LoNNe, ES1204) presents projects, raises awareness pertinent to ALAN, and stimulates an exchange of ideas and concepts [102,103].

Another form of knowledge exchange is via professional lighting and light pollution conferences. Professionals and academics across disciplines gather at these events to exchange content related to the latest developments within their profession. These events have become the locus of networking for likeminded and opposed individuals to discuss root problems and information related to their field of study. Today, the international conferences, as presented in Table 3, exchange an array of subjects with content strongly focused on topics relevant to the expertise of its audience’s disciplines (e.g., lighting professional conferences for lighting professionals, the lighting industry and manufacturers; light pollution conferences for academics, scientists, and experts on ALAN as a pollutant). These conferences adhere to their statement of purpose (e.g., lighting professional conferences present content relevant to the design practice, whereas light pollution conferences present content relevant to light pollution and ecological light pollution, and technical conferences present content relevant to technology and design focused on light as the primary technical solution).

Interdisciplinary participation rarely occurs due to the narrow and specific requirements and protocols of these conferences. An exception is the ALAN conference series, which is dedicated to examining all aspects of artificial light at night, including technology and design, biology and ecology, and health. In recent years, an initiative has begun to expand beyond the usual content and purpose statement in conferences and across disciplines (e.g., the presentation of empirical data on light pollution for lighting professional conferences or the presentation of a project or urban lighting application for light pollution conferences).

However, equitable transdisciplinary content is still required in order to evolve conferences into an interchangeable platform to exhibit the utility of the lighting practice and present evidence of artificial light as a pollutant. This commitment may provide more perspective and offer appropriate tactics that broaden the interpretation of lighting applications, as well as raise awareness about technological tools for urban environments.

Another step towards collaborative interactions between the practice and research of lighting is defining platforms and networks to make knowledge transferable. Due to globalization, the rise of networking has increased interactions via international conferences [104] and digital platforms [105], which render an opportunity to contextualize the exchange of ideas. These tools serve as knowledge transfer mediums to facilitate transdisciplinary and inclusive peer community praxis. They should be considered as a modus operandi to develop potential alliances that reduce cultural and societal ambiguities experienced by experts from different domains [106]. Again, the participation of the actors via knowledge transfer platforms may empower dialogue and blur the existing boundaries between the lighting practice, environmental research, and the policy-making of lighting, as well as the lighting industry.

## 5. Urban Lighting Research Transdisciplinary Framework—Actors, Framework, and the Four-Step Process

A theoretical principle of an organization towards sustainable lighting applications should encourage the interaction of four main domains: research, practice, production inputs, and policy-making and planning. The collaborative process between these four pillars can potentially facilitate the understanding of data as knowledge, the application of the acquired knowledge as standards to follow that may serve as guidance for the development of lighting equipment, and the development of methodologies to design lighting schemes protective of the night.

**Figure 2:**
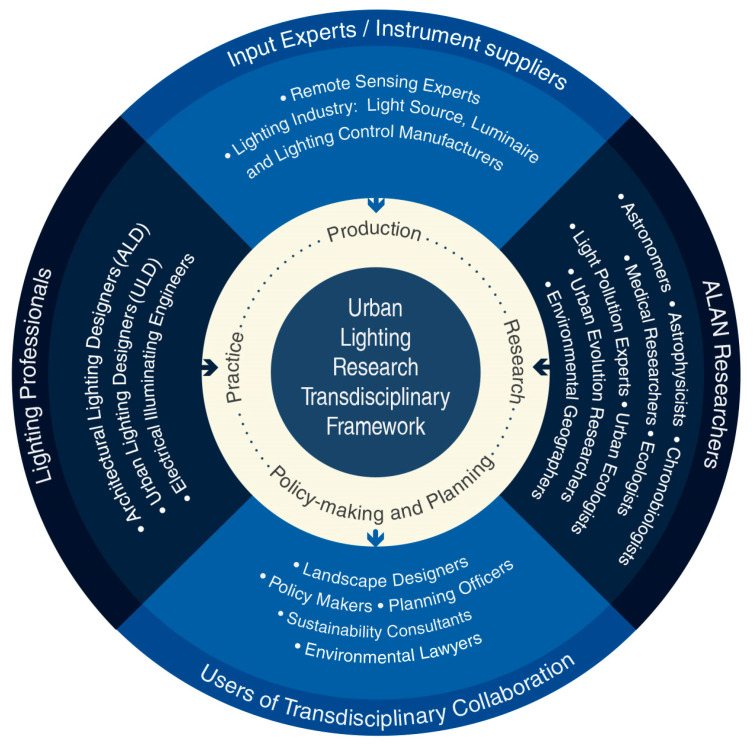
Proposal for an urban lighting research transdisciplinary framework. Source: authors’ own work.

To gain knowledge, in the context of ALAN as a pollutant from applied lighting technology, the Urban Lighting Research Transdisciplinary Framework (ULRTF) (Figure 2) proposes the participation of four domains and various actors to distinguish the practices and approaches of each field. It is of great importance to note that the lighting practice is not a uniform domain as it is characterized by different strategies and techniques. For instance, the main distinguishing difference between electrical illuminating engineers (e.g., street lighting engineers) and lighting designers (e.g., architectural lighting designers (ALD) and urban lighting design ULD) is their approach to lighting applications. Street lighting engineering is mainly focused on the application of visible luminaires to create homogeneous horizontal illuminance for pedestrian paths and vehicular circulation based on strict technical lighting standards (e.g., by the European technical standard EN 13201, CEN). In contrast, the approach of lighting design aims to create lighting for three-dimensional spaces that focus on light as a medium on a surface and not the luminaire. Additionally, the application of light by lighting designers integrates knowledge from different fields (e.g., architecture, urban planning, industrial and product design, landscape design, and perception psychology) to apply a modest presence of light on surfaces to sculpt volumes, enhance materials, create gradients, and reveal shadows in a determined space and context, as well make night-time spaces welcoming and user friendly.

ALAN experts (e.g., astronomers, astrophysicists, chronobiologists, medical researchers, ecologists, light pollution and ecophysiology experts, urban ecologists, urban evolution researchers, and environmental researchers) focus on lighting applications with scientific findings that assess the practice of lighting to consider light-sensitive species and ecosystems and to preserve night-time environments, and experts from the remote sensing field and instrument suppliers from the lighting industry (e.g., light source, luminaire and lighting control manufacturers) provide the necessary assistance on research, including light pollution data and the development of new lighting technologies, and testing tools to assess the application of lighting (e.g., [4,71,72,107]). However, one cannot forget about the users of this transdisciplinary collaboration, including urban planners, landscape designers, policy-makers, planning officers, sustainability consultants, and environmental lawyers, and their role in regulating fundamental aspects involving urban lighting masterplans and assessing the application of ALAN that balances the safety and security of users while considering nocturnality across nightscapes (e.g., [31,108,109,110]).

Table 3 presents a list of conferences as venues to exchange theoretic lenses on particular subjects of interest. These venues may offer an initial contact to a particular field of expertise considered foreign, to expand the scope of knowledge and jargon, to implement a preliminary social contact at an accepting level, to acquaint potential actors to establish ULRTFs, and to potentially generate new platforms (e.g., conferences and technical committees). These new platforms may serve as settings to implement ULRTF with effective communication and a periodic engagement of the actors in a collaborative fashion towards lateral thinking strategies and problem-solving mechanics rather than dwelling on monologues with essentially linear and vertical thinking [111]. ULRTF is a process to mindfully consider when attending these conferences as these may provide venues to meet potential actors to establish an opening to a collaboration framework [110].

Beyond the collaborative framework, it is also essential to establish a process with a series of key steps to align, structure, and develop sustainable short- and long-term goals. The collaborative development of steps and crucial processes can potentially favor the dissemination of diverse knowledge and the expertise of each domain to create crossover linkages of emerging approaches and procedures based on scientific practice curricula, and to provide a platform for emerging transdisciplinary professionals that attempt to involve scientific knowledge in their lighting practice. Table 4 presents an overview of a proposed four-stage collaborative process that aims to involve lighting professionals in urban lighting research.

## 6. Conclusions

The challenges of presenting collaborative perspective approaches for urban lighting research rely on decoding various opportunities and practices that include the social exchange of the actors, their professional motivation and purpose, and the collaborative nature to structure processes that encompass the diversity of ideas.

This insight article presents a brief overview of the rapid development and application of lighting technologies, which have unintentionally resulted in the widespread increase in unnatural brightness, and the application of colors of light across landscapes in cities and towns. Unfortunately, due to the problems distinctive to each separate field, the deeply rooted and different perspectives present in the principles of each profession, along with a lack of dialogue between them, this has resulted in a no-win/Gordian Knot situation. Therefore, this work proposes the application of the urban lighting research collaborative process as a community framework in order to identify the challenges that need to be addressed, the similarities that should be shared, the steps and procedures that must be followed, and the motivations and purposes that will help towards creating collective ecological awareness. Furthermore, this paper presents a new and potentially useful model for bringing different professionals together, where for the first time, lighting professionals as key players are introduced into the collaborative process.

In conclusion, the proposed ULRTF relies on various opportunities and practices to encourage the diversity of ideas in order to consider lighting parameters that operate ecologically and deliver safe and secure measures for users at night.

Moreover, this article also highlights the need for correctly designed urban lighting research, and it proposes the collaboration of knowledge between environmental experts, lighting professionals, and experts from other fields. Such a concept is envisaged as a continuous work in progress with periodic adjustments, with the understanding that the lighting practice is often ahead of the research field due to its knowledge of new technological developments in urban lighting. Research studies on ALAN continue to exponentially increase, which can provide an optimistic overview of the impending outcomes of the practice. The reasonable and logical next phase will be to translate the acquired knowledge of the research into practice to develop sustainable lighting concepts and techniques for future nightscapes [8] and to build an ecologically conscious and responsive society [48].

## Figures and Tables

**Table 1:** Examples of regulatory frameworks for urban lighting and light pollution based on [40,47,48] and references therein.

Category	Year	Name	Country of Origin
Guidelines	1997	CIE 126-1997. Guidelines for minimizing sky glow, by the International Commission on Illumination (CIE)	International
	2009	Artificial Light in the Environment Report, by the Royal Commission on Environmental Pollution	UK
	2017	CIE 150:2017. Guide on the Limitation of the Effects of Obtrusive Light from Outdoor installations, 2nd Edition, by the International Commission on Illumination (CIE)	International
	2018	IDA—International Dark Sky Community Program Guideline	International
	2019	The Australian Guidelines for Outdoor Lighting, by the Department of Environmental Protection	Australia
	2020	GN01-20. Guidance Note 1 for the reduction of obtrusive light 2020, by the Institution of Lighting Engineers (ILE)	UK
Procedures	2002	A handbook of practical guidelines for managing street lighting to minimize impacts on sea turtles, by the Florida Power Company, Coastal Roadway Lighting Manual	USA
	2011	Model Lighting Ordinance (MLO)—User’s Guide, by the International Dark Sky Association (IDA) and Engineering Society of North America	USA/International
	2018	Dark Sky Manual for Homeowners, by the Utah State Parks	USA
Standards and Codes	1989/2011	Flagstaff Outdoor Lighting Code	USA
	2003/2015	EN 13201-2: Road lighting—Part 2: Performance requirements, by the European Committee for Standardization (CEN)	Europe
	2014	EN 12464-2 Light and Lighting—Lighting of Work Places, Part 2: Outdoor Work Places, by the European Committee for Standardization (CEN)	Europe
	2020	ANSI/IES LP-11-20 Environmental Considerations for Outdoor Lighting, by the American National Standards Institute and Illuminating Engineering Society	USA/International
Legal Acts	1958	Flagstaff Lighting Ordinance	USA
	2006	Section 102 of the Clean Neighbourhoods and Environment Act (2005)	UK
	2007	Light Pollution Law concerning street lighting, facade illumination	Slovenia
	2011	Public Lighting decree in Berlin	Germany
	2011	Decree No. 2011-831 of 12 July, 2011 on the prevention and limiting of light pollution	France
	2012	Decree No. 2012-118 of 30 January, 2012 on the outdoor advertising, signs and signposting	France
	2013	Order of 25 January, 2013 on the night-time lighting of non-residential buildings in order to limit light pollution and energy consumption	France
	2018	Decree of 27 December, 2018 on the prevention, reduction and limitation of light pollution	France

**Table 2:** The evolution of outdoor lighting. Source: authors’ own work.

Figure 1	Description
(a)	During medieval times, most cities and towns were illuminated by night with point source illumination (e.g., candles placed adjacent to windows, lanterns positioned at a determined height in façades, and hand-held lanterns. Fire (e.g., present in candles and hand-held lanterns) was used as a beacon of light to help shape a sense of understanding of the urban realm after dusk.
(b)	Later, electrical engineers (EE) and illuminating engineers (IE) used gas and electrical light sources that included incandescent, low-pressure sodium (LPS), and high-pressure sodium (HPS) as point light sources for streets, roads, and paths to facilitate pedestrian and vehicular circulation [77]. Gas and electrical point sources of illumination were considered static as they lacked movement compared to previously applied light sources such as carried hand-held lanterns and gas-fueled fixtures that were frequently moved to the locations to provide visibility at night. Mounted lanterns on poles were introduced, and in the years following, the number of lanterns per fixed pole increased.
(c)	During the 20th century, IE and architectural lighting designers (ALD) favored an ensemble of lighting systems that vertically illuminated skyscrapers and tall buildings. Decorative advertising lighting systems also became popular. The economic growth of the post-war years instigated the application of emerging static point light sources that included fluorescent lamps (FL), ceramic metal-halides (CMH), and neon lamps to built environments that had formerly only been illuminated by incandescent, LPS, and HPS. Neon lamps were used for the lettering placed on the top of buildings to advertise brands, products, and locations, whereas incandescents were used to illuminate marquee signs and letters [78,79].
(d)	The end of the 20th century saw the introduction of urban lighting masterplans (ULM) by IE, EE, and ALD, and urban lighting planners (ULP) introduced ULM to revitalize the function of cityscapes and also to create a decorative appearance of cities at night as a symbol of economic growth and to boost tourism [65]. A wide range of approaches included functional point sources (e.g., for pedestrian and vehicular circulation), as well as decorative point sources (e.g., for the vertical illumination of historical buildings and landmarks for advertisement).
(e)	During the early years of the 21st century, a new emerging lighting technology called light emitting diodes (LEDs) became the preferred choice of technology by the IE, EE, ALD, ULP, and entertainment designers (EA) to illuminate built and natural landscapes (Figure 1e). LEDs offered low cost, easy application, and miniaturization for functional lighting for pedestrian and vehicular areas, decorative lighting for the enhancement of historical and modern buildings (e.g., skyscrapers, buildings, monuments, and landmarks), and static and dynamic lighting characteristics (rich in movement with changing colors and illuminance parameters) to the modern cityscape.

**Table 3:** Overview of lighting design and light pollution international conferences. Source: authors’ own work.

Conference	Main Topics	Focus
Light Pollution: Theory, Modelling and Measurements (LPTMM)	Astronomical observations, theoretical concepts and solutions, numerical modelling, and field campaigns on various topics that include the effects of atmospheric aerosols, clouds, terrain, and obstacles on light pollution, the impact of spectral and angular characteristics of light sources and reflecting surfaces, observational techniques instrumentation, data and products, and the design and evaluation of dark-sky-friendly lighting technologies, regulations, and outreach.	Scientific
European Symposium for the Protection of the Night Sky	Societal and cultural perspectives on light pollution, light pollution policy, citizen science resources, biodiversity and ecology, sustainable development, and outreach and initiatives.	Scientific
Artificial Light at Night (ALAN) Conference organized by the steering committee of the ALAN Conference	Technology and design, e.g., artificial lighting technology, architectural lighting, energy efficiency, outdoor lighting and street lighting; measurements and modelling, e.g., citizen science, human exposure, modelling, remote sensing, urban and pristine areas; society, e.g., economics, legislation, lighting governance, lighting conflicts, outdoor lighting applications, perceptions of the night and the preservation of natural areas, science and technological advancements, spatial security, and the history of lighting; biology and ecology, e.g., biodiversity, chronobiology, evolutionary adaptation, behavior, and food webs; health, e.g., circadian rhythm disruption, exposure to outdoor and indoor light, illness related to ALAN, and melatonin.	Scientific Practice-oriented
Professional Lighting Design Convention (PLDC) organized by VIA Verlag	Lighting application case studies, professional practice issues, philosophy and debate, office and retail lighting applications, plus workshops that include excursions to view illumination projects.	Design Practice-oriented
Enlighten Americas, Europe, Asia Conferences organized by International Association of Lighting Designers (IALD)	Art, e.g., communicating design and the artistic/creative side of lighting design (e.g., architectural lighting design (ALD) as an artistic medium), the artistic process in projects, the poetics of lighting design, artistic conceptions, future artistic trends, experimental design, and holistic approaches for lighting applications; science, e.g., lighting technology (sources, controls, fixtures, and software), development and trends, alternative energy sources (e.g., solar power), the internet of things (IoT), project management strategies, control integration, project case studies, cross-discipline topics and theory-based ideas, as well as the latest research; professional tools, e.g., factors and challenges of the practice, technology and creativity, business and social media, business and economy, trends and business, management tools, client management skills, contract negotiations, budgeting, marketing tools, as well as hiring and employee benefits.	Design Practice-oriented
Annual Illuminating Engineering Society (IES) Conferences organized by IES	Art, design, science and the research of lighting relevant to lighting professionals, educators, and related design disciplines.	Technology and Design
International Commission on Illumination (CIE) Conferences	The physiology of human vision, vision and quality of light and colored light, optical characteristics; light measurement methodologies, physical measurements of light, photometry and the spectrum of light sources; interior lighting and lighting design, quality of lighting, transportation and exterior applications; photobiology, photochemistry; energy efficiency, LED lighting, renewable energy sources; photobiological risk of artificial lighting.	Technology and Science

**Table 4:** Overview of a proposed four-step process for lighting professionals in urban lighting research. Source: authors’ own work.

Steps	Category	Description
Step 1	Problem Definition	State the problem by clearly defining questions, identifying the topics involved, and defining the existing solutions or case studies;
		Develop background research on the defined problems by searching scientific and lighting practice literature for comparison;
		Translate problem-driven topics into research questions and hypotheses.
Step 2	Research Design Development	Define the most appropriate properties of artificial lighting to be investigated during the research study;
		Determine the research procedure connected to artificial lighting;
		Define and evaluate the parameters of lighting samples used in the future study;
		Exchange and disseminate knowledge across disciplines related to the research;
		Identify of the limitations of the study.
Step 3	Conducting Research (Collecting and Analyzing Data)	Partner across organizational boundaries to assess and exchange on current (local and global) standards, regulations, and guidelines; to collect, exchange, and interpret data; and for the use of measuring equipment, light sources, luminaires, and lighting control types.
Step 4	Take Actions (Reporting Research Findings)	Translate and share research outcomes with the lighting practice in specific lighting publications;
		Co-write scientific research papers with other team members;
		Speak at lighting conferences and seminars;
		Develop guidelines and recommendations for the improvement of existing lighting approaches based on teams’ research study outcomes.
